# Coping Styles Utilized during Suicide and Sudden Death Bereavement in the First Six Months

**DOI:** 10.3390/ijerph192214709

**Published:** 2022-11-09

**Authors:** Sharna Mathieu, Racquel Todor, Diego De Leo, Kairi Kõlves

**Affiliations:** 1 Australian Institute for Suicide Research and Prevention, WHO Collaborating Centre for Suicide Research and Training, School of Applied Psychology, Griffith University, Brisbane, QLD 4122, Australia; 2 Slovene Centre for Suicide Research, Primorska University, 6000 Koper, Slovenia; 3 De Leo Fund, 35137 Padua, Italy

**Keywords:** grief, bereavement, coping, suicide, sudden death, stigma, shame, postvention

## Abstract

Individuals bereaved by suicide experience substantial emotional distress and are at risk for poorer mental health, substance use concerns, and suicidal behaviors. This study aimed to explore whether those bereaved by suicide reported different coping styles compared to those bereaved by sudden death in the first six months. It also aimed to determine whether a previous mental health diagnosis (PMHD) and experiencing stigma and/or shame impacted the utilization of adaptive and maladaptive coping. The sample was constituted by individuals bereaved by suicide (*n* = 142) compared to those bereaved by sudden death (*n* = 63), six months after loss. The study included immediate family members who were 18 years or older and understood the English language. After controlling for demographics there were no significant differences in coping styles between bereavement types. Regardless of bereavement type, having a PMHD was associated with increased avoidant and problem-focused coping, and stigma and shame were each associated with increased avoidant coping. Women were also more likely to report using adaptive coping. Findings demonstrate no difference by bereavement type and have implications for the tailoring of grief/postvention supports that are sensitive to perceived stigma/shame to better facilitate utilization of adaptive emotion-focused coping, particularly for men and those with pre-existing mental health problems.

## 1. Introduction

According to estimates from the World Health Organization, approximately 703,000 individuals died by suicide around the world in 2019 [1]. While each death is a preventable tragedy, the full magnitude of this public health issue is only fully realized when the far-reaching impact on family, friends, colleagues, and communities is also accounted for. Although difficult to determine, recent literature estimates that each suicide results in 135 exposed individuals [2], a third of which (e.g., family members, close relatives) are likely to experience ongoing moderate-to-severe emotional distress [3,4]. Furthermore, being bereaved by suicide, but also by other types of sudden death, can contribute to subsequent mental health problems and suicidal behavior [5,6,7]. Understanding the grief experiences and adaptive and/or maladaptive coping strategies used in the bereavement process is essential to inform and enhance adequate postvention interventions.

### 1.1. Bereavement

Traditionally, the grief process has been viewed as linear stages or phases that an individual progresses through before grief can be resolved; however, more recent theories have favored a fluid bereavement journey. The dual-process model of coping with bereavement by Stroebe and Schut [8,9] suggests there are two categories of stressors associated with bereavement: loss-oriented stressors (e.g., focus on the relationship with the deceased, circumstances of death), and restoration-oriented stressors (e.g., legal and financial issues resulting from the loss, new role/identity). Bereaved persons oscillate between loss- (e.g., relocating bonds) or restoration-oriented coping strategies (e.g., attending to life changes) depending on which stressors they are confronted with, where they are in their grief journey, as well as personal and cultural influences [9]. These coping strategies can be emotion- or problem-focused and be adaptive or maladaptive, depending on their appropriateness to the type of stressor and whether they serve to approach or avoid [9]. Regardless of theoretical conceptualizations, the process of bereavement is undoubtedly complex. Normative resolution takes time and relies upon adaptive coping [10], aided by sense-making and interpersonal connections [11] and managing the changing feelings, responsibilities and roles related to the loss [9]. Importantly, how someone initially copes with their grief may play a salient role in the trajectories of their bereavement [12].

### 1.2. Coping Strategies

Coping strategies can be categorized into three overarching styles: avoidant, active-emotion-focused and problem-focused coping [13]. Avoidant coping styles are typically viewed as maladaptive strategies used to avoid intolerable feelings and can include refusal to accept the loss and deep feelings of grief, alcohol or other substance abuse, blaming others, avoiding/denial of life and identity changes, or distraction [9,14,15]. A reliance on avoidant coping strategies is often linked with complicated grief and increased levels of depression and mental health problems [14]. Emotion-focused coping seeks to actively regulate one’s emotions, and can include talking about grief-related stressors, disclosing feelings, and interacting with others [13]. It has been suggested that grief focused interventions that directly target facing one’s intrusive thoughts and feelings can help to facilitate the grieving process in those with complicated grief symptoms [16]. Problem-focused coping can be described as active solution-making, such as creating a strategy for how to cope and taking action to improve the situation through planning [13,17]. It may help with certain practical stressors experienced during bereavement [9]. However, an over-reliance on problem-focused coping has been described as unhelpful, as bereavement is typically a situation outside one’s control [17].

### 1.3. Experiences of Shame and Stigma

There may be grief experiences unique to suicide bereavement that further impede the normative resolution of grief and promote the use of maladaptive avoidant coping strategies. For instance, while universal grief experiences (e.g., sadness) may be common to all bereavement, the experience of stigma and shame may be more likely in those who are bereaved by suicide and other forms of violent death [6]. Frequently, those bereaved by suicide report themselves as feeling judged, rejected, and misunderstood by their friends, families and community supports [18,19], and experience guilt, conflictual relationships, and concealment as a consequence of (perceived) stigma and shame [20,21]. The experience of stigma and shame may elicit maladaptive coping and may further isolate individuals from social support [22]. Indeed, individuals bereaved by suicide have reported that social support was often ill-timed and insufficient due to the stigma they experienced, and individuals therefore disengaged from their social networks [18,21,22]. For example, one large cross-sectional study found that around a third of relatives received no formal contact regarding the suicide of a family member who had been in touch with psychiatric facilities in the year prior to their death, and this was associated with potentially stigmatizing characteristics of the decedent such as criminal history, substance abuse, etc. [23]. This represents a considerable missed opportunity for assertive postvention support. Instead, individuals are often required to utilize alternative (solitary) ways to cope, including remaining silent and denying the cause of death [18,24]. This may contribute to disenfranchised grief: a grief that occurs when one cannot openly acknowledge or publicly mourn [25]. However, the distinction between suicide and other forms of bereavement is not clear-cut [26,27,28], with many studies conflicting in their findings regarding unique outcomes of suicide bereavement [6]. One explanation may be that differences may vary depending on which mode of death it is being compared to, such as expected versus other forms of sudden or violent death [6]. There are still relatively few studies which compare experiences of stigma and shame in those bereaved by suicide as compared to other forms of sudden death. However, it appears that while all forms of sudden death are associated with stigmatizing experiences, this may be heightened in those bereaved by suicide where additional impacts of self-stigma, shame and guilt are also experienced [29,30].

### 1.4. Aims

The use of adaptive coping strategies for regulating grief-related emotions throughout the bereavement process is important and may be influenced by underlying factors such as pre-existing mental health concerns [12]. However, it is unclear whether individuals recently bereaved by suicide engage in certain coping strategies over others as compared to those bereaved by other causes of sudden death, and whether the presence of underlying mental health conditions may impact the use of these strategies. Furthermore, unique grief experiences such as shame or stigma may adversely influence the use of coping strategies and ultimately grief trajectories for those bereaved by suicide. Therefore, the aims of the current study are to:

- investigate the coping styles of individuals bereaved by suicide as compared to sudden death in the first six months after the loss of their close relative;
- explore the impact of a previous mental health diagnosis (PMHD) on coping styles during early bereavement; and
- investigate how the experience of stigma and shame contribute to the coping styles of people bereaved by suicide.

## 2. Materials and Methods

### 2.1. Research Design

The current study forms part of a larger longitudinal investigation of suicide bereavement conducted over two years in Queensland, Australia [28,31,32]. A longitudinal prospective design was used to compare suicide bereavement to bereavement from other types of sudden deaths across three different time points: 6, 12 and 24 months after the death. The rationale to include sudden deaths as a comparison group was the similarity of the sudden and often unexpected nature of the death as opposed to long-term illnesses or other expected causes. To examine these associations in the early stages of bereavement, the current study utilizes data from the six-month time-point. Six months as an indicator of ‘early bereavement’ was deemed most appropriate by the research team and ethical approval committee in striking an important balance between recency to the highly distressing sudden loss (by suicide or other causes) and the burden of participation in an in-depth research study comprised of both qualitative and quantitative components.

### 2.2. Data Collection

The current study was approved by the Institution’s Human Research Ethics Committee (CSR/04/11/HREC). Inclusion criteria were: being close relatives and family members bereaved by a suicide or sudden death, aged 18 years old or older, able to speak and understand English, and, given that the larger longitudinal study from which the current study information was collected was centered on suicide and sudden death bereavement experiences in Queensland, the death had to have occurred in Queensland, Australia [28,31]. Participants were contacted 6 ± 1 months after the loss. Family members were defined as a spouse, de facto partner, adult child, parent, grandparent, grandchild, sibling, aunt, uncle, niece and nephew, and immediate in-laws. It should be noted that ‘children’ and ‘grandchildren’ refers to their kinship relationship type (not age) and were the adult offspring or family member of the decedents.

Those bereaved by suicide were identified through the Queensland Suicide Register, a real-time surveillance system. Clinical interviewers sent letters introducing the study, along with research information and consent forms to the identified individuals. Two weeks after the letters were dispatched, individuals were contacted by telephone to further introduce the study, obtain consent, and arrange a time and place for a six-month time-point interview. Of the participants that were interviewed (response rate 39.9%), 78.2% were interviewed via phone, and the remaining interviewed face-to-face either in their home or at the university.

Participants for the sudden death group were recruited through Queensland Office of State Coroners police forms. Only closed cases of “reportable deaths” of a sudden, violent, or suspicious nature as recorded by the Queensland Coroners Acts 2003 [33] were approached (i.e., not expected deaths due to long-term illness or age-related causes). Letters introducing the study, along with research information and consent forms, were sent to the reported next-of-kin of the deceased. All participants provided written informed consent to participate (response rate 16.1%). No phone calls were made for the sudden death group to collect consent. Half of the sudden death participants lost their loved one suddenly to diseases of the circulatory system (52.4%), followed by other external causes of death (25.4%; mainly transport accidents), sudden death by other medical causes (e.g., epilepsy, asthma; 9.4%), injury, poisoning, and other consequences of external causes (6.3%), and ill-defined and unknown cause of mortality (6.3%). The sudden death group only received responses from immediate family (partners, parents, adult children, and siblings). Therefore, for comparability of the two groups, all responses from non-immediate family members such as uncles, aunts, grandparents/children, and cousins were excluded from the suicide bereaved group.

Semi-structured interviews (~2.5 h duration) were conducted with participants by trained clinical interviewers with postgraduate health qualifications. A short introduction to the research was conducted at the beginning of the interview, and a debrief was conducted at the end of the interview to allow for participant’s concerns and questions. The interviews adhered to the following template: (1) a qualitative component—open-ended questions about the events leading to the death [34,35]; and (2) a quantitative component—sociodemographic information, medical and psychiatric history, including suicidal behavior of the deceased and bereaved, and post-event experiences measured with different validated scales [28,32]. A pilot study demonstrated the acceptability of the questionnaire and adequate procedures for recruitment [31] which are further described in more detail elsewhere [28]. The current study used quantitative information only.

### 2.3. Measures

In addition to basic demographic information for the bereaved participants (e.g., age, gender, type of kinship to the deceased person) and their deceased family member (e.g., age, gender, type of death—suicide or sudden), the participants also provided self-reported information on any previous mental health disorder diagnoses (PMHD) or treatment for they had received before the loss of their relative. The bereaved individuals also completed the following validated surveys:

The Grief Experience Questionnaire (GEQ) [36] is designed to measure typical and unique grief experiences. The version used for the current study was comprised of 40 items assessing the following grief experiences: somatic reactions, search for explanation, loss of social support, guilt, responsibility, rejection, stigmatization, and shame [31]. The subscales of stigmatization (e.g., “I feel uncomfortable revealing the cause of the death”; α = 0.81), and shame (e.g., “I feel embarrassed about the death”; α = 0.73) were used for the current study.

The BRIEFCope [37] is a 28-item survey which uses a 4-point Likert scale to assess different coping styles. Based on the conceptual and empirical literature [38,39], the items are grouped into the three coping styles: avoidant coping (α = 0.69), problem-focused coping (α = 0.82) and emotion-focused coping (α = 0.66).

### 2.4. Statistical Analyses

Analyses were conducted using the IBM SPSS, version 27 (IBM Corp, Armonk, NY, USA). Descriptive statistics, including means, standard deviations and frequencies were calculated. Dummy variables were computed for kinship type (partner, parent, adult child, and sibling). The Chi^2^ test and Fisher’s exact test were used to compare differences between suicide and sudden death bereaved using demographic variables. T-test and one-way ANOVA were utilized for preliminary comparison of coping styles across different groups. Hierarchical multiple regression analyses were conducted to identify factors associated with copying styles: avoidant coping, problem-focused coping, and emotion-focused coping. Age, gender of the deceased and of the bereaved, as well as kinship type, were entered in the first model as control variables. The independent variables, type of bereavement (suicide vs. sudden death), PMHD (yes vs. no), stigma (continuous), and shame (continuous) were entered step-by-step into regression models, respectively.

## 3. Results

### 3.1. Participants

The final sample included 142 suicide bereaved (average age: 52.7; SD: 11.6; female: 73.2%) and 63 sudden death bereaved (average age: 53.2; SD: 15.5; female: 69.8%) participants. For the suicide bereaved, the average age of the deceased person was 41.2 years (SD: 18.0; female: 19%) and in the sudden death group, 51.4 years old (SD: 17.1; female: 30.2%). Table 1 provides additional demographic characteristics. There were significant differences between bereavement types for kinship relationships and these were controlled for in the regression models.

Group differences by coping style are presented in Table 2. For bereavement type, there were no significant differences between avoidant and problem-focused coping; however, those bereaved by suicide reported significantly more emotion-focused coping than those bereaved by other sudden death. Men recently bereaved reported significantly less avoidant, problem-focused, and emotion-focused coping than women. Those with a PMHD reported significantly more avoidant, problem-focused, and emotion-focused coping than those without a PMHD. Finally, there were significant differences in problem-focused coping between different kinship groups. Spouses/partners reported the most problem-focused coping and offspring of the deceased reported the least. A previous study on the same sample found that after adjusting for kinship, gender, age, PMHD, self-harm (deceased, bereaved), current mental health symptoms and current suicidal ideation that those in the suicide bereavement group reported experiencing significantly more stigma and shame than the sudden death bereavement group at six months [32].

### 3.2. Avoidant Coping

Model 1 was significant, with the age and gender of the bereaved contributing significantly to the prediction of avoidant coping (*F*(6,197) = 2.64, *p* = 0.017; Table 3). Bereavement type was entered into model 2 with no significant change (*F*(1,196) = 1.05, *p* = 0.306), meaning there was no significant difference between people bereaved by suicide and those bereaved by other sudden death. The addition of PMHD accounted for 2.8% additional variance in avoidant coping (*F*(1,195) = 6.19, *p* = 0.014); individuals with a PMHD reported significantly higher levels of avoidant coping. Stigma significantly accounted for 7.9% of unique variance in model 4 (*F*(1,194) = 18.75, *p* < 0.001); greater experience of stigma was associated with increased use of avoidant coping. Shame was added into the final model (*F*(1,193) = 5.70, *p* = 0.018), accounting for 2.3% of unique variance, where an increased experience of shame related to an increase in avoidant coping. In addition, having a PMHD and increased experience of stigma remained significant in the final model.

### 3.3. Problem-Focused Coping

Model 1 predicting problem-focused coping during bereavement was significant (*F*(6,197) = 6.25, *p* < 0.001; Table 3), with gender and age of the bereaved, and being an adult child of the deceased, significantly contributing to problem-focused coping. Bereavement type did not significantly account for any additional variance in model 2 (*F*(1,196) = 0.77, *p* = 0.381). PMHD significantly accounted for 2% unique variance in model 3 (*F*(1,195) = 4.71, *p* = 0.031). Stigma did not account for any significant variance in model 4 (*F*(1,194) = 2.365, *p* = 0.127) and neither did shame in model 5 (*F*(1,193) = 0.50, *p* = 0.479). However, PMHD, gender and age of the bereaved and being an adult child of the deceased (compared to partners/spouses) remained significant predictors of problem-focused coping in the final model. More specifically, being female, younger-aged, and having a PMHD increased the probability of problem-focused coping, and being an adult child of the deceased was associated with decreased problem-focused coping.

### 3.4. Emotion-Focused Coping

Model 1 was significant in predicting emotion-focused coping (*F*(6,197) = 3.52, *p* = 0.002; Table 3), where gender and age of the bereaved was significantly associated with emotion-focused coping. Bereavement type did not account for any significant variance in model 2 (*F*(1,196) = 2.69, *p* = 0.103); neither did PMHD in model 3 (*F*(1,195) = 2.45, *p* = 0.119). Stigma significantly accounted for 2.3% of unique variance towards emotion-focused coping in model 4 (*F*(1,194) = 5.32, *p* = 0.022). Shame did not significantly predict any unique variance in the final model (*F*(1,193) = 3.11, *p* = 0.08). Higher levels of stigma and female gender of the bereaved significantly predicted more emotion-focused coping in the final model.

## 4. Discussion

The current study focused on coping style differences among individuals bereaved by suicide and by other sudden death six months after the loss of a close relative. In addition, the impact of a previous mental health diagnosis, stigma, and shame on coping styles were analyzed. The literature has shown mixed results regarding the experiences and outcomes of suicide bereavement as compared to other types of loss [26,40]. In the current study, there were no significant differences between the two bereavement types and their use of avoidant, problem-focused or emotion-focused coping styles, suggesting similar coping styles in the early stages of bereavement after adjusting for age, kinship type and gender (of the bereaved and deceased). A recent study found that those bereaved by suicide reported significantly higher avoidant coping than those bereaved by combat loss, yet there were no significant differences when compared to those bereaved by accidents, nor were there any differences for self-reported active/problem or emotion/supportive coping in either of the bereaved groups [41]. However, time since the loss varied from one to twelve years [41], whereas the current study analyzed coping and grief experiences only in the first six months, which provides a timely insight into this critical initial bereavement period.

There was no significant difference in the prevalence of a PMHD between the two bereavement groups. Nevertheless, individuals with a PMHD (regardless of bereavement type) reported significantly higher levels of avoidant and problem-focused coping, while there was no significant difference for active emotion-focused coping. This is consistent with the literature, whereby avoidance of emotions and internal sensations (i.e., experiential avoidance) has been linked to several mental health conditions, including depression and anxiety [42]. It is less clear why a PMHD may relate to increased problem-focused coping. One explanation could be that those with a self-reported PMHD onset before the loss of their loved one may have previously sought help for their mental health concerns (hence receiving a diagnosis) and are thus already more active/experienced in help-seeking and more able to draw upon problem-focused coping strategies during the early stages of their bereavement. Another explanation could be that previous experience of mental health concerns makes bereavement more complicated and therefore a reliance on a greater number of coping strategies that are arguably easier to apply are employed in an effort to manage. That is, focusing on practical matters or avoiding facing painful feelings may be ‘easier’ in more complex or highly painful situations of bereavement (e.g., [15]). In the current study the problem-focused coping subscale included items related to active coping (i.e., trying to actively remove a source of stress), planning (i.e., considering practical steps to take), instrumental support (i.e., seeking practical advice), and religion (i.e., turning to religion/religious activities). While problem-focused coping is generally considered more adaptive than avoidant strategies, an overreliance on problem-focused coping may be less desirable in coping with bereavement whereby many situational elements are outside one’s control [17]. Instead, according to the dual-process model of grief, adaptive grief resolution over time relies upon the appropriate use of both emotion-focused and problem-focused coping to manage the fluctuation of loss-oriented and restoration-oriented stressors, and that flexible oscillation between healthy coping strategies is required [9]. That is, the coping strategies of the bereaved can be both adaptive and maladaptive depending on how rigidly one is relied upon above others and whether they are appropriate for the source of stress [9].

In our study, in the final models after accounting for all variables of interest and demographics, bereaved women were more likely to use problem- and emotion-focused adaptive coping than bereaved men, but there were no differences for avoidant coping. Gender differences in coping with grief have long been discussed [43], and recent research implies that gender may influence the trajectory of ‘prolonged grief’ over time, with men experiencing more pronounced grief initially which decreased and vice versa for women (there were no differences between genders for more stable and resilient grief profiles [44]). However, there may be few differences regarding actual self-reported symptoms of prolonged grief experienced between genders [45]. The current preliminary findings have implications for the tailored provision of postvention and grief support. For example, while men and women demonstrated similar levels of avoidant coping strategies (after accounting for all variables of interest and demographics), the tendency for men in this study to report less utilization of adaptive emotion- and problem-focused coping strategies in the initial six-month period following loss may correspond to patterns observed in previous studies (e.g., [44]) and it may be particularly useful for practitioners to focus on enhancing adaptive coping strategies in men during this earlier period, which may serve to facilitate grief resolution and contribute to the prevention of suicidal behaviors and other adverse outcomes [6]. Naturally, this relies upon people getting access to timely and appropriate postvention supports which may be impeded by (real or perceived) experiences of stigma and shame [23,46,47]. However, further research is required to determine gender differences in perceived experiences of stigma and shame during bereavement from suicide and sudden death, and to develop and evaluate effective postvention supports that are sensitive to gender differences in coping [48].

### 4.1. Stigma and Shame

Irrespective of bereavement type, greater self-reported experiences of stigma and shame were each associated with increased avoidant coping in the initial six months of bereavement. This is consistent with the literature, as stigma and shame have been associated with behaviors of withdrawal and secrecy in both suicide and sudden death bereaved populations [19]. More recently, stigma has been described in relation to increased self-reported thwarted belongingness (i.e., alienation from others) in those bereaved by suicide, which, consistent with the interpersonal theory of suicidal behavior, would be associated with suicidal ideation over time [49]. Interestingly, in our study, those who reported greater experience of stigma also reported increased emotion-focused coping (this association was non-significant for shame). Neither shame nor stigma were associated with problem-focused coping.

To date, there is limited research on how coping strategies interact with experiences of stigma and shame during early bereavement. Future research is required to replicate these findings. Nevertheless, these results challenge the notion that stigmatization after suicide or sudden bereavement may hinder one’s ability to utilize adaptive coping due to isolation, avoidance, rejection, and fear of discussing the death. One explanation could be that when informal support systems of the bereaved fail to provide adequate assistance, bereaved individuals may seek support elsewhere (e.g., counsellors or support groups). In a recent Lithuanian study, those bereaved by suicide and who sought professional support reported higher levels of stigmatization and guilt than those who did not seek professional support [50]. On the one hand, mental health professionals may provide a more appealing environment to express vulnerable emotions and share personal grief stories to process their loss than informal supports who may be perceived as judgmental or unhelpful (e.g., [18]). On the other hand, by engaging with professional supports, individuals may gain insight into the impacts of societal stigma through the psychoeducation and interaction provided by a supportive professional. A second explanation may relate to the use of the Internet as an alternative source for social support. Studies have shown that individuals bereaved by suicide make social media posts and email friends and family about the loss [51], and they are increasingly accessing blogs, websites, and social networking platforms to interact with other bereaved individuals and memorialize their loved ones [52]. When individuals utilize the Internet in this manor, it has been demonstrated that not only did it remove some fear of stigmatization, but it also helped heal their sorrow [51]. A final explanation could be that the experience of stigma makes grief more complicated, and thus, a greater number of coping strategies are relied upon (adaptive or maladaptive).

### 4.2. Limitations

The current study was cross-sectional and had more than double suicide bereaved individuals (*n* = 142) compared to sudden death bereaved individuals (*n* = 63). Therefore, the sudden death group may not accurately represent full unique variance, resulting in possible missing effects. There were also group differences in the (low) response rates, with those bereaved by suicide having a higher response rate (39.9%) than other causes of sudden death (16.1%). The low response rates likely introduce some bias into the current findings and restrict generalizability. It is understandable that in the early stages of bereavement there may be a general lack of interest in participating in research, hence the low response rates. Reasons for non-participation were available only for the suicide bereaved group and included inability to contact the individual, the individuals not wishing to discuss their bereavement, or the individual being too busy. Differences in recruitment procedure may explain why those bereaved by suicide were more likely to respond to the study invitation. Other possible reasons could be that the research team were from a suicide prevention research institute, which may have unintentionally influenced responses. Furthermore, given the additional layers of shock and challenges with sense-making that may be experienced or perceived following a suicide (as opposed to a shocking sudden death that can be ‘explained’ by a medical reason), there may have been more motivation to engage with research aimed at furthering our understanding and informing postvention supports. Additionally, the sudden death group was comprised of deaths of heterogenous causes, with the majority comprised of sudden deaths of the circulatory system (e.g., heart attacks) and accidental deaths (e.g., car accident). There may be differences in how experiences of stigma and shame are associated with different coping styles across these subgroups; for example, sudden death causes where a sense of guilt or blame can be assigned (subjectively or objectively through the legal system) as opposed to medical causes. Unfortunately, due to small numbers it was not possible to analyze differences by subgroups and this remains an important area for future research.

Although the larger longitudinal study did include measurements assessing concurrent distress and mental health outcomes [28], the current analysis did not include them, as it was outside of the scope of the study aims. However, by demonstrating that a PMHD was significantly associated with increased avoidant coping, it posits the question as to whether the increased use of avoidant strategies due to a PMHD contributes to negative or positive mental health outcomes in individuals bereaved by suicide and sudden death in the early bereavement period or over time. The current analysis did not include information about individuals’ current treatment for their PMHD. Therefore, the current study cannot definitively suggest that having a PMHD promotes an effect on coping styles, or whether it is the type of treatment that may have been received (either previously and/or during the initial bereavement period). These questions should be investigated in future research.

Finally, there are always challenges in selecting survey measures that are valid, robust, and sensitive to change over time. For the current study, the use of a coping style survey designed for use in response to stressful life events may not comprehensively capture all facets of coping during the complex period of bereavement. Nevertheless, the survey measure has been used in previous bereavement studies (e.g., [38,39]). Furthermore, the shame and stigma subscales of the grief experiences questionnaire may have also minimized complexities in relation to self-directed versus external stigma, as well as internal versus external shame. The items in the current study mostly reflect external experiences of grief-related stigma, and the grief-related shame items arguably have some conceptual overlap with avoidant coping strategies.

### 4.3. Implications

Current findings provide new information regarding the early bereavement experiences of those bereaved by suicide and other sudden death and have important implications for promoting adaptive coping for grief resolution over time. Postvention service delivery frameworks should be tailored to the various levels and needs of support [53]. Postvention workers, therapists, and grief counsellors working with those recently bereaved by suicide or sudden death should be mindful of underlying mental health conditions and what impact these may have on the grief experience and coping strategies utilized. While the current study included different previous diagnoses, the majority of existing PMHD in the current sample were anxiety disorders and depression. The finding that experiences of stigma and shame were each associated with increased avoidant coping is also important for mental health practitioners to note. Not only are real or perceived experiences of stigma and shame distressing and isolating, but avoidant coping has also been implicated in disenfranchised and complicated grief [25]. Clinicians should be mindful of a client’s overreliance on maladaptive avoidant coping strategies in the early stages of bereavement, particularly in those with previous experiences of mental health concerns, and in situations of suicide or sudden death where stigma and shame are present. One promising avenue that requires further research is interventions for bereaved individuals focused on mindful self-compassion, which directly targets feelings of shame, stigma, or guilt as well as mental health and wellbeing, and adaptive emotion regulation and coping [54,55]. Regardless of content and delivery, postvention and grief supports should be developed and guided by the involvement of those with lived experience of suicide loss so as to be maximally sensitive to their needs, such as practical support in the early period, perceived experiences, as well as the impact of features such as gender and kinship [21,26,27,56]. It is also crucial that services are assertive, and people bereaved by suicide or sudden death are identified and followed up with quickly to minimize the potential interference of stigma and shame on help-seeking behaviors and other coping strategies.

## 5. Conclusions

The current study demonstrated that both bereavement groups reported similar coping styles six months after the loss of a close family member. The results indicated that having a PMHD increased both avoidant and problem-focused coping in both the suicide and sudden death bereaved. Grief-related experiences of both stigma and shame were associated with increased use of avoidant coping strategies. Men were less likely to utilize adaptive coping strategies such as active emotion-focused and problem-focused coping. The utilization of adaptive coping strategies is amenable [57], and there is a need for timely and appropriate postvention and grief supports that are effective and tailored to the needs and experiences of those with lived experience of suicide [53] and sudden loss.

## Figures and Tables

**Table 1:** Demographic characteristics of the sample, including gender of the deceased.

	Bereavement Type					
	Suicide (*n* = 142)		Sudden Death (*n* = 63)		Chi^2^_(df)_	*p*
	*n*	%	*n*	%		
Gender of the bereaved					0.25_(1)_	0.616
Male	38	26.8	19	30.2		
Female	104	73.2	44	69.8		
Gender of the deceased					3.11_(1)_	0.078
Male	115	81.0	44	69.8		
Female	27	19.0	19	30.2		
Kinship to the deceased					13.19_(3)_	0.004
Spouse/partner	41	28.9	31	49.2		
Parent	72	50.7	17	27.0		
Adult child	16	11.3	11	17.5		
Sibling	13	9.2	4	6.3		
PMHD ^1^					0.14_(1)_	0.713
No diagnosis	104	73.2	48	76.2		
One or more	38	26.8	15	23.8		
Depression	24	16.9	13	20.6		
Anxiety	11	7.7	5	7.9		
Bipolar	4	2.8	1	1.6		
Other	10	7.0	2	3.2		
Ethnicity						
Caucasian/White	134	94.4	60	95.2		1.000 ^2^
Aboriginal	3	2.1	1	1.6		1.000 ^2^
Non-Caucasian	5	3.5	2	3.2		1.000 ^2^
Interview type						
Face-to-face	31	21.8	7	11.1	3.32_(1)_	0.068
Over the phone	111	78.2	56	88.9		

^1^ People could self-report more than one PMHD; for later analyses this was recoded into ‘yes, any PMHD’ or ‘no diagnosis’. ^2^ Fisher’s exact test was performed.

**Table 2:** Group differences by coping styles.

	Coping Style								
	Avoidant			Problem-Focused			Emotion-Focused		
	*M (SD)*	*t/F*	*p*	*M (SD)*	*t/F*	*p*	*M (SD)*	*t/F*	*p*
Bereavement		1.09	0.279		0.87	0.384		2.22	0.027
Suicide	13.82 (3.91)			21.01 (5.69)			27.46 (5.21)		
Sudden Death	13.17 (4.05)			20.25 (5.90)			25.67 (5.59)		
Gender of bereaved		−2.54	0.012		−3.55	<0.001		−3.66	<0.001
Male	12.51 (3.86)			18.54 (5.69)			24.75 (5.42)		
Female	14.05 (3.92)			21.64 (5.55)			27.74 (5.14)		
Kinship		1.17	0.324		4.79	0.003		0.88	0.453
Spouse/partner	14.30 (4.10)			22.63 (5.36)			27.08 (5.55)		
Parent	13.41 (3.77)			20.26 (5.73)			27.29 (5.15)		
Adult child	13.00 (3.61)			18.33 (5.72)			25.44 (5.83)		
Sibling	12.94 (4.70)			19.71 (5.59)			26.47 (5.16)		
PMHD		2.96	0.003		2.74	0.007		2.14	0.033
Yes	15.02 (3.91)			22.62 (5.00)			28.25 (3.78)		
No	13.17 (3.87)			20.13 (5.87)			26.41 (5.76)		

**Table 3:** Regression models for each coping style.

	Avoidant				Problem-Focused				Emotion-Focused			
	β	sr^2^	∆R^2^	R^2^	Β	sr^2^	∆R^2^	R^2^	Β	sr^2^	∆R^2^	R^2^
**Model 1**				0.074 *				0.160 ***				0.097 **
Age (Bereaved)	−0.20 *	0.03			−0.24 **	0.05			−0.17 *	0.02		
Gender (Deceased)	<0.01	<0.01			0.03	<0.01			−0.02	<0.01		
Gender (Bereaved)	0.14 *	0.02			0.20 **	0.03			0.22 **	0.04		
Kinship (Parent)	−0.03	<0.01			−0.11	0.01			0.09	0.01		
Kinship (Adult child)	−0.15	0.02			−0.30 **	0.07			−0.12	0.01		
Kinship (Sibling)	−0.06	<0.01			−0.09	0.01			0.02	<0.01		
**Model 2**			0.005	0.079 *			0.003	0.163 ***			0.012	0.109 **
Age (Bereaved)	−0.19 *	0.03			−0.24 **	0.04			−0.16 *	0.02		
Gender (Deceased)	0.01	<0.01			0.04	<0.01			−0.01	<0.01		
Gender (Bereaved)	0.14	0.02			0.19 **	0.03			0.22 **	0.04		
Kinship (Parent)	−0.05	<0.01			−0.13	0.01			0.06	<0.01		
Kinship (Adult child)	−0.15	0.02			−0.30 **	0.07			−0.12	0.01		
Kinship (Sibling)	−0.07	<0.01			−0.10	0.01			<0.01	<0.01		
Bereavement type	0.07	<0.01			0.06	<0.01			0.12	0.01		
**Model 3**			0.028 *	0.108 **			0.020 *	0.183 ***			0.011	0.120 **
Age (Bereaved)	−0.19 *	0.03			−0.23 **	0.04			−0.15 *	0.02		
Gender (Deceased)	0.02	<0.01			0.04	<0.01			−0.01	<0.01		
Gender (Bereaved)	0.12	0.01			0.18 *	0.03			0.21 **	0.04		
Kinship (Parent)	−0.04	<0.01			−0.12	0.01			0.06	<0.01		
Kinship (Adult child)	−0.15	0.02			−0.30 **	0.07			−0.12	0.01		
Kinship (Sibling)	−0.06	<0.01			−0.09	0.01			0.01	<0.01		
Bereavement type	0.07	<0.01			0.06	<0.01			0.11	0.01		
PMHD	0.17 *	0.03			0.14 *	0.02			0.11	0.01		
**Model 4**			0.079 ***	0.186 ***			0.010	0.193 ***			0.023 *	0.144 ***
Age (Bereaved)	−0.08	<0.01			−0.20 *	0.03			−0.10	0.01		
Gender (Deceased)	0.05	<0.01			0.05	<0.01			0.01	<0.01		
Gender (Bereaved)	0.10	0.01			0.17 *	0.02			0.20 **	0.03		
Kinship (Parent)	−0.03	<0.01			−0.12	0.01			0.07	<0.01		
Kinship (Adult child)	−0.08	<0.01			−0.28 **	0.06			−0.09	0.01		
Kinship (Sibling)	−0.02	<0.01			−0.08	<0.01			0.03	<0.01		
Bereavement type	−0.01	<0.01			0.03	<0.01			0.07	<0.01		
PMHD	0.15 *	0.02			0.13 *	0.02			0.09	0.01		
Stigma	0.33 ***	0.08			0.11	0.01			0.17 *	0.02		
**Model 5**			0.023 *	0.210 ***			0.002	0.195 ***			0.014	0.157 ***
Age (Bereaved)	−0.07	<0.01			−0.20 *	0.03			−0.10	0.01		
Gender (Deceased)	0.04	<0.01			0.06	<0.01			0.01	<0.01		
Gender (Bereaved)	0.08	0.01			0.18 *	0.03			0.21 **	0.04		
Kinship (Parent)	−0.03	<0.01			−0.12	0.01			0.06	<0.01		
Kinship (Adult child)	−0.11	0.01			−0.27 **	0.05			−0.06	<0.01		
Kinship (Sibling)	−0.04	<0.01			−0.07	<0.01			0.04	<0.01		
Bereavement type	−0.02	<0.01			0.03	<0.01			0.08	0.01		
PMHD	0.14 *	0.02			0.14 *	0.02			0.10	0.01		
Stigma	0.23 **	0.03			0.14	0.01			0.24 **	0.04		
Shame	0.18 *	0.02			−0.06	<0.01			−0.14	0.01		

* *p* < 0.05, ** *p* < 0.01, *** *p* < 0.001; NB: For kinship, the reference group is ‘spouse/partner’.

## Data Availability

De-identified study data can be made available upon reasonable request.
